# Virtual individual cognitive stimulation therapy (V-iCST): Mixed methods feasibility randomised controlled trial

**DOI:** 10.1016/j.ijchp.2024.100523

**Published:** 2024-11-22

**Authors:** Esther K. Hui, Victoria Tischler, Gloria H.Y. Wong, Luke Gibbor, Chiara Lousley, Georgia Bell, Maria Jelen, Tiffeny James, Rob Saunders, Charlotte Stoner, Elizabeth Sampson, Aimee Spector

**Affiliations:** a Department of Mental Health of Older People, Division of Psychiatry, University College London, London, United Kingdom; b School of Psychology, The University of Surrey, Guildford, United Kingdom; c Department of Social Work and Social Administration, The University of Hong Kong, Hong Kong SAR; d Research Department of Clinical, Educational and Health Psychology, University College London, London, United Kingdom; e Faculty of Education, Health and Human Sciences, University of Greenwich, London, United Kingdom; f Marie Curie Palliative Care Research Department, Division of Psychiatry, University College London, London, United Kingdom

**Keywords:** Cognitive stimulation, Dementia, Psychosocial intervention, Teletherapy

## Abstract

**Objective:**

Cognitive Stimulation Therapy (CST) is a dementia intervention shown to improve cognition and quality of life (QoL). Previous research on individual CST delivered by family carers showed no significant improvements in people with dementia. We aimed to evaluate the feasibility and acceptability of Virtual Individual Cognitive Stimulation Therapy (V-iCST) delivered by healthcare personnel.

**Methods:**

Mixed methods feasibility randomised controlled trial. Thirty-four participants were randomly allocated to either 14 sessions of twice-weekly V-iCST (*n* = 17) or treatment as usual (*n* = 17) delivered over seven weeks. We assessed cognition, QoL, communication, and depressive symptoms pre/post-treatment. We conducted semi-structured qualitative interviews with participants and carers (*n* = 15) following V-iCST, analysed with thematic analysis.

**Results:**

High levels of attendance, adherence, completion of outcomes, and moderate fidelity. There were no significant between-group changes, but there was a positive trend in cognition. Qualitative findings suggested that V-iCST was valued and convenient but can evoke negative emotions.

**Conclusions:**

V-iCST was feasible and acceptable. Preliminary data indicate that V-iCST delivered by healthcare personnel might meet a critical gap through increasing access to those who cannot or prefer not to attend in-person CST/groups. The need for remote treatments and CST being the main psychosocial intervention emphasizes the need for definitive trial.

Dementia, a public health priority and a significant cause of disability, affects over 55 million people worldwide (World Health Organization, 2021). Group Cognitive Stimulation Therapy (CST) is an established, cost-effective group intervention for people with mild to moderate dementia and the only non-pharmacological intervention recommended by the UK National Institute of Health and Care Excellence (NICE) to improve cognition, independence, and well-being. A typical CST programme consists of 14, 45 min sessions twice a week over 7 weeks (National Institute for Health and Care Excellence, 2006, 2018; Spector et al., 2003). Since its development in the UK, group CST has been researched or delivered in around 28 countries. The global CST literature continues to confirm benefits to cognition, with several studies showing improvements in QoL and mood. Qualitative studies have complemented trial findings, demonstrating that CST enhances confidence and improves mood (Gibbor et al., 2021).

The COVID-19 pandemic highlighted the need for remote interventions, but even before the pandemic, not all were suitable for, and accessed CST. Orrell et al. (2017) developed individual CST (iCST) to increase accessibility and person-centred care for those with sensory impairments and no transport provision. Unlike CST, where trained professionals delivered the therapy, iCST used carers and offered 75 sessions, three times a week (Orrell et al., 2017). While PwD in Orrell et al. (2017) had no improvements, an iCST study that mirrored elements of group CST—non-family member-led and offering 14 sessions—indicated a 5-point increase in Alzheimer's Disease Assessment Scale – Cognitive Subscale (ADAS-Cog) (Gibbor et al., 2021). Four points are considered clinically significant (Food & Drug Administration, 1989; Schrag & Schott, 2012).

As a response to the pandemic, a virtual version of iCST was developed in collaboration with researchers developing virtual CST and evaluated as a mixed methods case series study in Hong Kong (Hui et al., 2022; Perkins et al., 2022). They found that V-iCST was feasible and acceptable for Hong Kong Chinese in a small case series with no attrition and high attendance (100%). There was also a slight positive trend in cognitive function as the effect size of the improvements were comparable to past CST studies (Woods et al., 2023). Qualitative findings suggested that it was convenient, stimulating, and enjoyable. Building upon the findings of the Hong Kong study, the current study aimed to:

• Assess the feasibility and acceptability of the V-iCST programme for people with dementia (PwD)
• Explore potential impact of V-iCST on cognition, mood, and quality of life
• Assess the feasibility of conducting a full randomised controlled trial evaluating V-iCST

## Methods

### Overview

This study was a single-blind feasibility RCT of 14 sessions of V-iCST compared to TAU over seven weeks for people with mild to moderate dementia. It was registered on clinicaltrials.gov (NCT04828434). University College London Ethics Committee approved this study (reference: 17127.002).

### Recruitment and setting

We recruited participants from April to December 2021. We approached national charities (i.e., Age UK), community groups (e.g., Camden Carers), care homes (e.g., Royal Star and Garter), Join Dementia Research network for recruitment (Join Dementia Research, 2017) in the UK. We recruited participants from across the UK, including both community and care settings, to ensure diversity. People who met the inclusion criteria and expressed interest in participating were contacted via phone or videoconferencing for eligibility screening. If eligible, they provided written informed consent electronically.

### Participants

The inclusion criteria were: 1) a clinical diagnosis of dementia; 2) mild to moderate dementia determined using Montreal Cognitive Assessment – BLIND [MoCA-BLIND]>2; 3) 18 years or older; 4) could communicate in English; 5) capacity to consent; 6) ability to complete assessments; 7) access to videoconferencing; 8) consent to videorecording. We excluded participants who could not provide informed consent for the trial, or who had illness and/or disability affecting their participation (UK Department of Health, 2005). No power calculations were conducted. Informed by a previous feasibility study of iCST (Gibbor et al., 2021), we anticipated a sample size of 34 would provide sufficient information to address the objectives.

### Randomisation or allocation groups

Enrolled participants’ names were converted to participation identification number (PIN) before baseline assessments. A researcher not directly involved with data collection or facilitation conducted the randomisation on a 1:1 ratio using a web-based tool. All sixteen participants who received V-iCST were recruited for qualitative interviews.

### Intervention

The V-iCST intervention involved 14, 45-min sessions over seven weeks. Session material was adapted from Gibbor et al. (2021) for online delivery, following the same themes (see Table 1). Facilitators were PhD students, clinical psychology trainees, and psychology graduates who attended a one-day CST training course and/or had delivered iCST or CST previously. Participants were provided with a V-iCST package with guidance on “How-to-use Zoom”, key principles of V-iCST and a list of resources and optional materials to bring to sessions.

**Table 1:** V-iCST Session Themes.

Session	Theme
1	Physical games
2	Life history
3	Sounds
4	Childhood
5	Food
6	Faces/scenes
7	Word association
8	Being creative
9	Categorising objects
10	Orientation
11	Using money
12	Number games
13	Word games
14	Thinking cards

### Treatment as usual (TAU)

TAU referred to the usual care participants received, which includes stimulating tasks and activities (e.g., playing bingo and doing crossword puzzles) offered by day care centres and online support groups. We collected data on medication and general service use for all participants, to enable understanding of what TAU might look like.

### Feasibility and acceptability

Feasibility of recruitment and retention was assessed by 1) recruiting a target sample of 34 participants in 10 months; 2) retention rates of ≥75% at nine-week follow-up; 3) attendance and retention rate of at least 60% at follow-up; 4) and any negative or adverse event related to the intervention. Acceptability of randomisation was evaluated by whether there was a difference in the two groups in terms of number of dropouts. We used the assessor's perception of group application at follow-up to analyse the acceptability of blinding.

We developed a fidelity measure alongside this trial based on the key principles of CST and core intervention components of each session using a framework for measures for complex interventions and previous measures (Walton et al., 2019; Walton et al., 2020). Ten questions were on the key principles of CST and five on core intervention components. A total fidelity score (fidelity score/total score of the checklist x 100%) was calculated for each V-iCST session. Eighty percent-100% was considered high fidelity, 51%−79% as moderate, and ≤50% as low (Borrelli, 2011). All therapy sessions were video recorded. Fidelity was evaluated by facilitators completing the fidelity measure immediately after each session.

### Exploratory outcome measures

We conducted baseline assessments within the week before the commencement of V-iCST sessions, and post-test assessments within the week following the last session. Validated measures were adapted for virtual use, e.g., images were used instead of physical objects, and they were administered via videoconferencing. Carers completed proxy measures one working day before the session or during the meeting.

#### Cognition

1) MoCA-BLIND is a brief screening tool, adapted from MoCA for people with visual impairments (Wittich et al., 2010). It contains five domains: attention, language, abstraction, memory and orientation. This measure was chosen for the ease of virtual delivery, and as it is more sensitive in detecting changes in those with mild cognitive impairments than the gold standard for cognitive assessment, MMSE (Nasreddine et al., 2005). 2) ADAS-Cog was selected as it is more sensitive than the MoCA-BLIND with additional questions on short-term memory (Rosen et al., 1984). It is a 11-item self-completed scale assessing memory, language, and praxis. This validated instrument has good test-retest reliability (*r* = 0.93) and internal consistency (Cronbach's α=0.81), and it is frequently used in dementia drug trials (Weyer et al., 1997).

#### Quality of life

QoL-AD evaluates QoL in dementia with decent validity (*r* = 0.14 to 0.39), and good internal consistency (Cronbach's α=0.82–0.90) (Logsdon et al., 2002; Thorgrimsen et al., 2003). It contains self-completed and proxy-completed (family or professional carer) components, and the combined score is used. Each item is rated on a 4-point Likert scale (“poor”, “fair”, “good”, and “excellent”). There are 13 items, and total scores range from 13 to 52, with higher scores reflecting better QoL.

#### Depression

GDS-15 is a dichotomous self-completed scale that screens for depressive symptoms (Yesavage & Sheikh, 1986). A validation study shows that GDS-15 can differentiate non-depressed and depressed participants with a high correlation (*r* = 0.84, *p* < 0.01) and is sensitive to PwD (Feher et al., 1992; Yesavage & Sheikh, 1986).

#### Carer/proxy measures

##### QoL

See above section for details. Carers completed QoL-AD as an online survey or an interview during the pre/post-test assessment (Logsdon et al., 2002).

##### Communication

HCS is a 12-item standardised measure evaluating conversation, awareness, knowledge, and communication (Holden & Woods, 1995; Strøm et al., 2016). Responses range from 0 to 4, with a maximum of 48 points; lower scores indicate better communication abilities. It has been validated for PwD and has good internal consistency (Cronbach's α=0.94) and test-retest reliability (*r* = 0.71).

### Qualitative interviews

One author (CL) conducted semi-structured interviews via Zoom after the therapy programme with participants. The interviews lasted 30 to 60 min each. Some were interviewed independently, and others were accompanied by their carers. We developed the interview guides using the five-stage framework by Kallio et al. (2016) and previous qualitative research on V-iCST (Hui et al., 2022). The questions covered the five consolidated framework of implementation research (CFIR) domains: intervention characteristics, outer setting, inner setting, characteristics of individuals and process of implementation (see Table A.3) (Damschroder et al., 2009). The aim of the interviews was to evaluate acceptability of V-iCST, but we used the CFIR framework as identifying barriers and facilitators could support wider implementation in future.

### Analysis

We analysed all available data using the intention-to-treat principle. We used analysis of covariance to estimate between group differences in V-iCST and TAU for PwD at follow-up and baseline. The model was adjusted for baseline scores, and we calculated effect sizes using Cohen's *d*.

Adhering to the intention-to-treat principle, we imputed missing data with multiple imputation by chained equations using predictive mean matching 20 times. We excluded participants without carers from analyses that required a proxy. Sensitivity analyses were used to reanalyse the outcome to assess the robustness of the findings. We analysed the imputed data without adjustments for baseline characteristics and conducted complete case analyses with and without adjustments for baseline imbalances.

CL analysed the qualitative data using reflexive thematic analysis approach to explore the participant experience of V-iCST (Braun & Clarke, 2006). As CL transcribed the interviews by hand, she became familiar with the data (stage 1). Subsequently, she semantically coded the interviews (stage 2) by reading and re-reading the transcripts, generating initial themes (stage 3). Themes were refined (stage 4), defined and named (stage 5), and written up (stage 6) in discussion with 2 members of the author team (AS and VT).

### Anticipated risks and modifications to manual

We expected few adverse events in this trial because there were no documented harmful side effects in past CST and iCST trials (Spector et al., 2003). All serious adverse events were planned to be reported to the PI.

Facilitators completed session rating forms with three feedback questions after each session. Using this information, facilitators modified the manual and resources to produce a final draft.

## Results

**Fig. 1:**
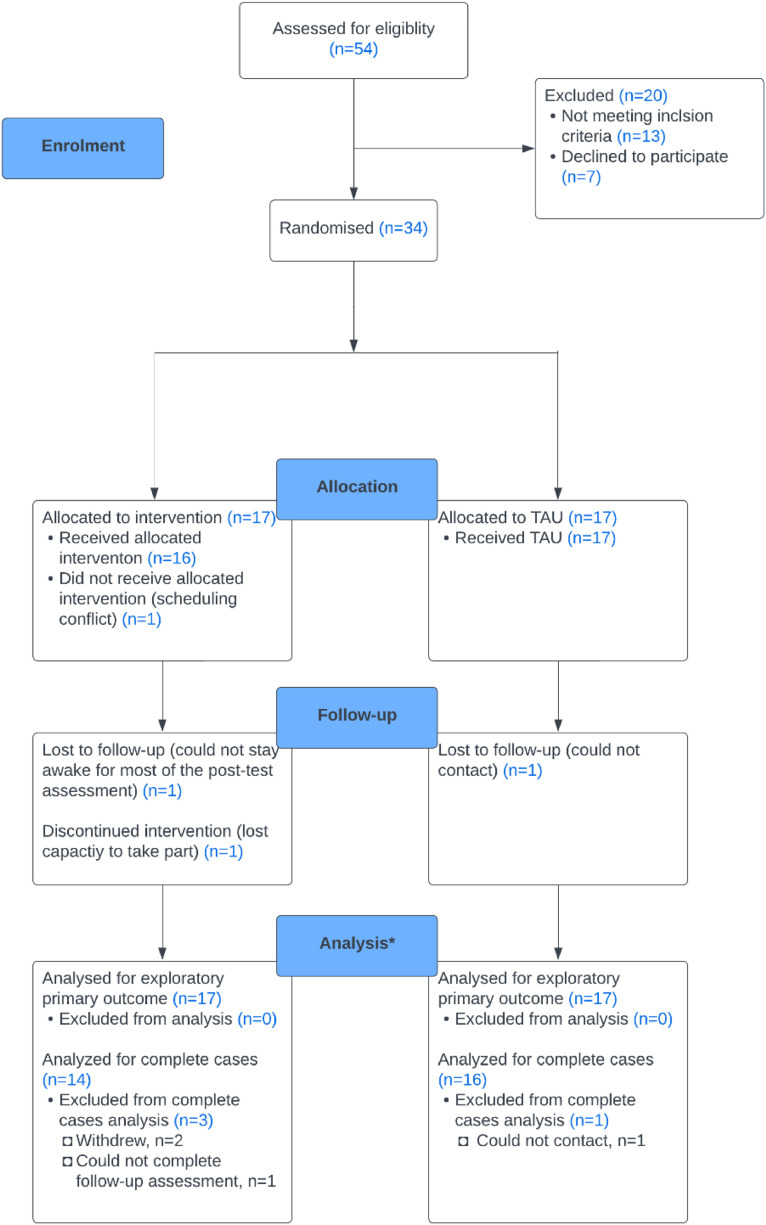
Flow of participants through study.

*Note.* Participants without carers at baseline.

We included 34 participants, allocating 17 to the treatment group and 17 to TAU. All were recruited from Join Dementia Research and Camden Carers. Thirty participants were assessed at follow-up, including 14 V-iCST and 16 TAU participants. Fig. 1 shows the flow of participants. Of the 54 participants screened, seven refused to take part, and 13 did not meet the inclusion criteria.

Table 2 shows the participants’ demographics. Ages ranged from 55 to 91 years (*M*=71.6, SD=8.66), where six had early onset dementia. Most participants were male (70.6%) and were on anticholinesterase medication at baseline (79.4%). All participants took part online in their own homes using computers or tablets. Randomisation achieved good balance in terms of sociodemographic and clinical characteristics for PwD. There were no statistically significant differences between the two groups at baseline except in the MoCA-BLIND.

**Table 2:** Baseline Characteristics of PwD. Values are Numbers (Percentages) Unless Stated Otherwise.

Characteristics	Total (*n* = 34)	V-iCST (*n* = 17)	TAU (*n* = 17)
Mean (SD) Age (years) (range)	71.61 (8.66) (55–91)	70.29 (8.45) (55–91)	72.94 (89.1) (58–87)
Sex			
Male	24 (70.6)	14 (41.2)	10 (29.4)
Female	10 (29.4)	3 (8.8)	7 (20.6)
Ethnicity			
White	33 (97.1)	17 (50.0)	16 (47.1)
Asian or Asian British	1 (2.9)	0 (0.0)	1 (2.9)
Marital Status			
Married	27 (79.4)	14 (41.2)	13 (38.2)
Cohabiting or civil partnerships	2 (5.9)	1 (2.9)	1 (2.9)
Widowed	2 (5.9)	2 (5.9)	0 (0.0)
Separated	2 (5.9)	0 (0.00)	2 (5.88)
Divorced	1 (2.9)	1(100)	0 (0.0)
Educational Level			
School leaver (14–16 years old)	5 (14.7)	2 (5.9)	3 (8.8)
School leaver (18 years old)	4 (11.8)	3 (8.8)	1 (2.9)
Further education (vocational qualifications, i.e., NVQ)	8 (23.5)	4 (11.8)	4 (11.8)
Higher education (i.e., BSc/BA equivalent)	13 (38.2)	6 (17.7)	7 (20.6)
Postgraduate education (e.g., MA, MSc, PhD, etc.)	4 (11.8)	2 (5.9)	2 (5.9)
Taking anticholinesterase inhibitors			
Yes	27 (79.4)	14(41.2)	13 (4.4)
No	7 (20.6)	3 (8.8)	4 (1.4)

**Fig. 2:**
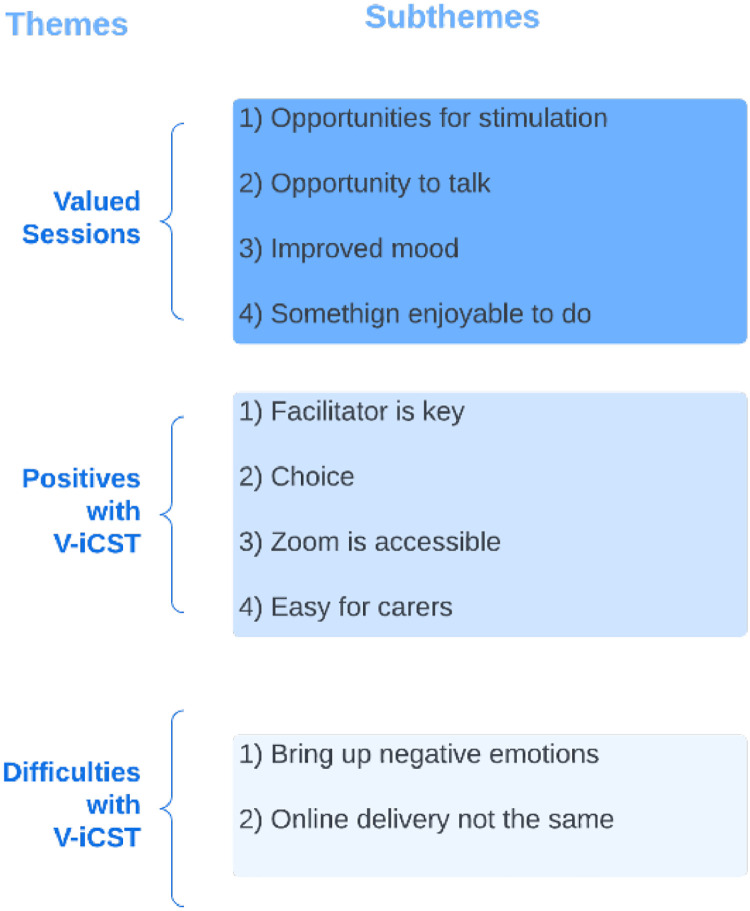
Identified themes and subthemes.

Of the participants in the qualitative sample, 15 PwD agreed to participate, including one that dropped out of the study seven carers took part in the interviews, six were spouses and one was an adult child. Over half (57.14%) were female. All identified as White, and the average age was 72.8 years. The thematic analysis generated ten themes and 10 subthemes (see Fig. 2).

## Feasibility and acceptability findings

### Recruitment and retention

We recruited a target sample of 34 in seven months. Retention was high (88%) at nine-week follow up (see Fig. 1). One participant in the treatment group withdrew from study after randomisation due to scheduling conflict. Two participants in the treatment group did not complete the post-test assessments: one lost the capacity to participate and dropped out after attending five therapy sessions, the other could not engage in assessments. In TAU, we could not contact on participant at nine-week follow up after two attempts.

### Randomisation and blinding

The low drop-out rate, predominately in the treatment group (see Fig.1), suggests that randomisation was feasible. In terms of maintaining blindness, almost half of the researchers (46%) perceived the group allocation correctly, 39% were neutral and 14% reported incorrect allocations.

### Acceptability of the intervention

The attendance rate of 90.3% of all participants in the treatment group indicated high adherence and acceptability of the interview. Females (100%) attended better than males (88%). However, attendance was similar in other clinical and demographic factors, such as age and use of medication. Fifteen completed all sessions, two completed less than half, and one dropped out during post-test assessment. No unexpected adverse events occurred.

### Theme 1. Valued sessions

Participants valued V-iCST session and indicated that sessions were stimulating and created space for them to be intellectually challenged. Stimulation was seen as beneficial, and some described this continued after the sessions.

P3: “I found them stimulating and left the sessions still with ideas buzzing around my head that I was thinking about later on, so I found them very worthwhile”

Sessions gave participants a chance to talk to someone outside of their family, where they could share their opinion and feel listened.

P5: “I guess for the individual [it] makes them feel worthwhile, that their view is heard or their opinion is being sought, or just being listened to”

Having a positive impact on mood during or after the V-iCST sessions formed the subtheme “improved mood.”

P5: “it was uplifting, so you know afterwards and ruing I feel very happy, so that's nice”

### Theme 2. Positives with V-iCST

#### Facilitator is key

Participants and carers highlighted how developing a good relationship with the facilitator was critical. Key characteristics of the facilitator included being personable and professional.

PC2: [I] think the charm of the facilitator was the key[…]me and my wife quite enjoyed talking to her.

Interviewer: do you think it helps that a psychologist conducted it? because before a previous version of this was done by carers, like family carers?

P3: it wouldn't have worked for me…when it is someone you don't know it's quite liberating, it gives you more confidence actually.

#### Choice

Participants stated the choice they were given during the sessions gave them a sense of agency. Without this choice, it might have been harder for some to participate.

P11: having been given options for things helped[…]because if you are only faced with one you think god I don't know anything about that[…]if you felt at a disadvantage because there was nothing there that you could talk about that would have been awful, but because you had the choice it made it much nicer

#### Zoom is accessible

Participants thought the videoconferencing platform was convenient as it was easier and more affordable to attend at home, and it also meant that V-iCST could be available to more individuals as it is not limited to those who can attend a community venue.

P9: “it is not just the question of the meeting, it is the getting there and getting back, and since I've stopped driving that becomes a problem. I don't like travelling on buses and I'm a distance away from the train station, so that would present a problem, so the problem gets relaxed or solved by using taxis, but that costs money. So, Zoom is much easier, I am in my own environment…”

### Theme 3. Difficulties with V-iCST

Some participants reported difficult aspects of sessions, which were mitigated by facilitators.

#### Sessions can bring negative emotions

Participants were often anxious before the first session as they were unsure what to expect and worried about how they would perform.

P11: “I was a bit nervous for the first time […] because I wasn't sure what the format was going to be and I didn't know whether it was going to be so difficult that I had to keep saying I don't know, and that would have been devastating. I would have hated that.”

Anxiety was also present throughout the sessions for some because they found it difficult to confront their own cognitive decline, which perhaps would be otherwise avoided or not realised. This realisation could be difficult. Both frustrating and embarrassing when participants struggled with activities.

P8: “It does make people feel less [less of themselves] and think that's important that you realise that […] being challenged and perhaps you are not having such a great day it doesn't make you feel so great […] and I think as a grown woman it's not such a nice place to sit.

P3: “every now and again you are confronted with your decline and as you go down this [dementia] journey, you are on, they are quite sad moments, quite profound moments when you can no longer do something you could a few months ago, it's a shame.”

#### Online delivery not the same

Most participants said they would prefer face-to-face over online sessions because elements of communication can be missed during online sessions, such as body language, posture, like someone leaning in, due to only being able to see shoulders and above on Zoom. There is a lack of opportunity to prepare and leave the house, which can be stimulating.

P13: “there is no doubt whatsoever, I would prefer face-to-face, but obviously it's not possible sometimes.”

P2: “much better in person of course. Because all sorts of information is delivered that you don't know about, it comes when you are with someone, well I don't need to say anymore.”

### Fidelity

The facilitators submitted self-reported fidelity ratings for sessions. While all participants (*n* = 17) consented to be video recorded, there was missing data. There were 51 (21%) missing checklists: 24 due to the participants’ absence, 19 from checklist formatting issues, and eight from facilitators not completing it. See Table A.6 for full breakdown of scores per facilitator and the fidelity measure. Overall, the mean total fidelity score was 27.5 points out of 35 points (78%, SD=3.90, range=16–35), indicating moderate fidelity (Borrelli, 2011).

### Secondary outcomes

This feasibility study was insufficiently powered to draw conclusions, and there were no statistically significant differences between groups for all exploratory outcomes. See Table 3 for the imputed data, adjusted for baseline outcome measures. While changes in ADAS-Cog were in the direction of improvement, other reported outcomes were not.

**Table 3:** Comparison of the V-iCST and TAU Group Changes from Baseline and at 9 weeks, Adjusting for Baseline Outcome Measures (Imputed Data).

	V-iCST at 9 weeks	TAU at 9 weeks	V-iCST change from baseline	TAU change from baseline	Difference in scores (95% CI), p-value	
Self-reported (*N* = 34)						
ADAS-Cog	13.0 (7.88)	16.7 (6.90)	−4.18 (7.88)	−0.41 (6.90)	−3.77 (−9.12; 1.59)	0.159
MoCA-BLIND	13.5 (3.32)	13.3 (3.11)	−0.11 (3.32)	−0.344 (3.11)	0.230 (−2.11; 2.56)	0.841
QoL-AD (self-reported)	37.7 (3.56)	38.9 (3.51)	1.23 (3.56)	2.39 (3.51)	−1.17 (−3.67; 1.33)	0.346
GDS-15	3.3 (1.56)	3.20 (15.94)	−0.48 (1.56)	−0.56 (15.94)	0.078 (−1.06; 1.21)	0.888
Proxy reported (*N* = 32)						
QoL-AD (combined)	37.3 (3.37)	38.2 (3.16)	1.03 (3.37)	1.89 (3.16)	−0.87 (−3.19; 1.46)	0.449
QoL-AD (proxy)	35.6 (4.16)	36.4 (3.84)	0.13 (4.16)	0.934 (3.84)	−0.80 (−3.64; 2.038)	0.565
HCS (proxy)	10.3 (3.66)	8.62 (3.35)	1.25 (3.56)	−0.48 (3.32)	1.66 (−0.89; 4.21)	0.191

#### Cognition

Comparison of change in ADAS-Cog scores, adjusting for baseline, showed a mean difference of 3.77 points (95% CI=−9.12;1.59, *p*=0.159, Cohen's *d*=0.509) in favour of V-iCST. In the sensitivity analysis, where the model did not include adjustment for baseline scores, results were similar, with a mean difference of 2.94 (95% CI=−8.57;2.87, Cohen's *d*=0.386).

Findings from other sensitivity analyses were similar to the primary analysis—the imputed and adjusted model. For complete cases, there was a mean difference of 1.18 (95% CI=−3.50;1.14, *p*=0.308, Cohen's *d*=0.385) between groups for cognitive function, measured by ADAS-Cog, when adjusting for baseline differences (see Table A.4 for details).

For the MoCA-BLIND, there were no statistical or clinical differences between the two groups pre and post-test (MD=0.23, 95% CI=−2.11;2.56; *p*=0.841, Cohen's *d*=0.017). The adjusted model for MoCA-BLIND showed a reduction in scores in the treatment group but not in the control group at nine weeks (see Table 4).

**Table 4:** Comparison of the V-iCST and TAU Group Changes from Baseline and at 9 weeks, Without Adjustments (Observed Data).

	9 weeks (*n* = 17)				Change from baseline (*n* = 17)				Difference in scores (95% CI), p-value	
	Missing	V-iCST	Missing	TAU	Missing	V-iCST	Missing	TAU		
ADAS-Cog	3	11.7 (9.82)	1	17.9 (12.54)	3	−1.68 (2.95)	1	−0.501 (2.94)	−1.18 (−3.50; 1.14)	0.287
MoCA-BLIND	3	15.1 (4.87)	1	11.9 (4.22)	3	−0.713 (2.05)	1	−0.125 (2.78)	−0.839 (−1.18; 0.653)	0.361
QoL-AD (self-report)	3	37.2 (5.06)	1	39.3 (3.05)	3	0.643 (4.01)	1	1.44 (4.18)	−0.794 (−2.28; 3.87)	0.601
GDS-15	3	3.43 (1.91)	1	3.13 (1.71)	3	0.357 (1.95)	1	−0.250 (1.81)	0.607 (−2.01; 0.80)	0.383
QoL-AD (combined)	5	36.9 (4.68)	1	38.6 (3.14)	5	0.472 (3.65)	1	1.19 (3.23)	−0.72 (−1.96; 3.39)	0.588
QoL-AD (proxy)	4	34.9 (4.88)	1	37.3 (5.39)	4	0.077 (4.48)	1	0.688 (2.75)	−0.61 (−2.16; 3.39)	0.655
HCS (proxy)	4	8.85 (4.83)	1	9.12 (6.23)	4	1.46 (3.13)	1	−0.438 (2.90)	1.88 (−0.53; 4.29)	0.102

#### Quality of life

Intervention and TAU groups reported higher QoL scores at the 9-week follow-up, however, improvements were non-statistically significant between group and the effect was small in size. Absolute improvement in QoL was greater in TAU, and the mean difference was −0.87 points (95% CI= -3.19;1.46, *p*=0.449, Cohen's *d*=0.263) when adjusting for baseline outcome measures. This finding was consistent when the QoL-AD was analysed as separate self-reported and proxy measures.

#### Depression

At baseline, participants in both V-iCST (*M*=15.79; SD=13.91) and TAU (*M*=18.47; SD=10.94) did not meet the cut-off score of four points for depression. There was no significant difference in depression scores post-treatment between groups in adjusted models (MD=0.078, 95% CI=−1.06; 1.12, *p*=0.888, Cohen's *d* = 0.007).

#### Communication

Adjusted models for communication showed a mean difference of 1.66 points change in scores (95% CI=−0.89; 4.21, *p*=0.191, Cohen's *d*=0.502), favouring the control group. While the changes were non-significant, there was a medium effect, indicating that V-iCST may not benefit communication.

### Other

No items in all outcome measures reported missing data. Missing data were solely from participants lost at the 9-week follow up. No adverse effects were reported.

In general, results from the sensitivity analyses were the same as the primary analysis—non-statistically significant between-group differences for all outcomes. See Table 4 for complete case analyses. Additional analyses including the models with and without baseline adjustments for the observed and imputed data at nine weeks and changes from baseline are found in Table A.4–5.

We modified the preliminary manual to ensure that it was more user-friendly for virtual delivery after the trial commenced. For example, virtual galleries, Google Map's augmented reality, online computer games should only be used with good internet connection. We also added extra resources to accompany most sessions; more content on food and money from different countries, ensuring culturally appropriate materials for a wide range of PwD. See (Hui, 2022) for the final version of the manual.

## Discussion

### Key findings

Results suggest that V-iCST was feasible and acceptable for those with mild to moderate dementia. Meeting the recruitment and retention targets with high attendance in therapy sessions indicated that it could be evaluated in larger trials. A moderate self-completed fidelity rating suggests the intervention was delivered as planned, yet improvements could be made. No missing assessment items indicates the selected outcome measures appeared suitable. None of the between-group changes in score differences in the exploratory outcomes were statistically significant. However, there was a slight positive trend in cognition.

Qualitative interviews also indicated that V-iCST was acceptable as participants valued the sessions. While most still preferred face-to-face therapy, they recognised that it is not always possible and stated the V-iCST was enjoyable and they looked forward to the sessions.

#### Interpretations

This trial was not sufficiently powered to draw any conclusions on the exploratory outcomes. While the difference in V-iCST and TAU were statistically non-significant, the small to medium effect size observed in the current trial (Cohen's *d*=0.386–0.509) is similar to the previous CST studies (Woods et al., 2023) and the 14-session iCST study (Orrell et al., 2017) and higher than the definitive iCST trial (Cohen's *d*=0.30) (Gibbor et al., 2021). Since cognition is one of the main CST outcomes, the effect size of it in this study is within range of what is important. The qualitative theme “opportunity for stimulation” also suggests that participants felt mentally challenged during and after sessions. With sufficient power, the differences in means might reach statistical significance like previous definitive trials. However, small samples can have larger effect sizes because of sampling, focused intervention delivery and other biases.

Compared to carer-led iCST trial, this study had better adherence and fidelity (Orrell et al., 2017), which may affect treatment efficacy. Several factors, including having a trained facilitator and shortening the dosage of the programme (with the carer-led trial having 75 sessions) may have contributed to this difference.

Some family members may lack the skills to deliver therapeutic sessions or not view this as their role (Yates et al., 2015). The American Medical Association (2022) stated that treating family members poses challenges, including concerns about objectivity, patient autonomy and informed consent. These issues were echoed in our qualitative results which emphasized the importance of the facilitator being a personable and professional psychologist.

#### Dose and content

Attendance and retention rates indicate that the original CST dosage used in this study as more feasible than the dosage in Orrell et al. (2017). Carers in the past iCST trial complained that it was difficult to fit 30-min therapy sessions three times a week (Orrell et al., 2017).

The V-iCST content differed from the iCST trial as there were only 14 sessions, not 75. Similar to Gibbor et al. (2021), we selected the most enjoyable sessions based on past field-testing findings (Yates, 2016). Participants are more likely to engage in activities they find stimulating or interesting, and this could have contributed to higher adherence.

### Strengths and limitations

Moderate fidelity indicates room for improvement in intervention delivery, however including a fidelity measure was a strength as this had not been done in previous studies. The high number of ‘not applicable’ (19.7%; 37/188) responses suggest that facilitators might not have been aware that the key principles were mandatory for every session.

While the intervention was evaluated in both urban and rural areas across the UK, the sample lacked diversity in terms of ethnicity and education, because only one person was non-white and 23% received higher education. This limits generalisability.

The main activities in the CST programme might have been too straightforward for the high functioning participants in the current study, creating a ceiling effect. Participants in the CST programme had mostly moderate dementia at baseline (MMSE=14.2) (Spector et al., 2003). This study sample's baseline mean score was 17.5 on the MoCA-BLIND (MMSE=23). While facilitators attempted to make activities more challenging, feedback from the session rating forms and fidelity ratings suggest sessions were still too simple at times.

Over 23.5% of the participants had rare forms of dementia, making findings less representative and perhaps different from the previous CST and iCST trials. In the UK, 35,000 people have a rare dementia (3.7% of all dementia cases) (Alzheimer's Research UK, 2022). While past trials recruited participants with all types of dementia and none examined whether subtypes respond to cognitive stimulation differently, most had Alzheimer's disease, vascular dementia, or mixed dementia (Orrell et al., 2014; Woods et al., 2012).

We only recruited PwD with access to videoconferencing and the internet. Participants with technological devices are likely to have higher SES (McMaughan et al., 2020) and is unclear whether this teletherapy would be feasible for those who do not have access to technology.

Since assessors guessed a higher number of groups correctly, some participants may have been unblinded during the post-test assessment. However, this must be interpreted with utmost caution due to the limited sample size. Larger trials are required to explore this further.

As V-iCST is proposed as a potentially more accessible alternative to other in-person interventions, including CST and iCST, an economic evaluation would be useful. It is a limitation that this current study does not include one, and it would be essential to incorporate it in future fully powered RCTs.

### Clinical implications

Having two participants living alone indicates that some PwD can participate in V-iCST independently. This adds an advantage as it widens the scope of people who can take part and could potentially carers to have some respite.

The virtual nature of the intervention allowed PwD who require or prefer remote access to receive treatment. Past studies have suggested that those with sensory impairments, no or limited transportation (i.e., those in remote areas of the UK) had difficulties in attending in-person and/or group therapies, such as CST (Orrell et al., 2017). This study showed that PwD could join online with carers' support. This could therefore potentially bridge treatment gaps, offering a viable alternative treatment option.

According to the cost analyses conducted for the iCST and CST trials, it is apparent that individual psychosocial interventions like iCST are more expensive than group CST (Knapp et al., 2006; Orgeta et al., 2015). More time and staff are required as facilitators can only treat one person instead of up to eight PwD at a time. With in-person iCST, therapists need to travel to each participant's home. Delivering V-iCST could be more cost-effective than in person treatment, as facilitators would not need to travel. However, future cost evaluations are warranted.

### Implication for future research

Larger trials are needed to evaluate the treatment efficacy of V-iCST, and its impact on a broader sample of people. Should V-iCST be effective, future studies can explore whether trained laypeople could deliver the programme.

Future trials should consider using volunteer or professional therapists. All facilitators in the current trial were psychology graduates with no clinical qualifications. Using volunteers without professional qualifications could make the intervention cost-effective. They may also be more objective than carers as they are not personally involved with PwD.

Given that the current sample lacked diversity, future studies should consider recruiting from minority ethnic group third sector organisations across diverse communities in the UK. Having a diverse ethnic and socioeconomic participants would help us understand the effectiveness of V-iCST in different populations. Researchers should consider providing technological devices (i.e., computers and/or tablets) with high-speed broadband access to ensure that participants with lower socioeconomic status have equal opportunities to receive treatment.

This was the first time this V-iCST fidelity measure was used for a trial. The fidelity measure requires further refinement. Researchers should present clear instructions and thorough training on the fidelity measure with guidance to the facilitators that key principles are critical to the delivery of every session. Future research could also include independent researcher ratings on a subset of therapy sessions to evaluate inter-rater agreement between them and the self-completed measures. It could also explore whether adherence is affected by the usability of technology.

Since there was no missing data from outcome measures, the assessments appeared suitable and indicated that the ADAS-Cog, as in other CST trials; could be used as the primary outcome for future trials.

## Conclusion

Overall, this 14-session virtual version of iCST was feasible and acceptable considering attendance, retention, fidelity, adverse events and preliminary outcomes. The findings suggest that there was a slight positive trend in cognitive function. V-iCST may be an alternative to in-person CST or iCST, which could potentially benefit those without access to treatment during the pandemic and beyond. We did not find improvements in QoL, communication, and mood in PwD. However, larger trials are warranted to draw firm conclusions as this trial was not powered to detect statistical significance.

## Funding

This research did not receive any specific grant from funding agencies in the public, commercial, or not-for-profit sectors.

## Declaration of competing interest

The authors declare that they have no known competing financial interests or personal relationships that could have appeared to influence the work reported in this paper.

## Appendix Supplementary materials

Image, application 1
