# Supplementary material for: Virtual individual cognitive stimulation therapy (V-iCST): Mixed methods feasibility randomised controlled trial
Source: Int J Clin Health Psychol. 2024 Nov 22;24(4):100523. doi: 10.1016/j.ijchp.2024.100523 (PMC11625133; doi:10.1016/j.ijchp.2024.100523)
Supplement: Supplementary file 1 [file mmc1.docx]

# Virtual Individual Cognitive Stimulation Therapy (V-iCST): mixed methods feasibility randomised controlled trial

**Appendix**

**Table A.1.** Baseline Data of PwD. Values are in Numbers (Percentages) Unless Stated Otherwise

| **Outcome measure** | **V-iCST** | **TAU** |
| --- | --- | --- |
| Self-reported (N=34) |  |  |
| ADAS-Cog | 15.79 (13.9) | 18.47 (10.9) |
| MoCA-BLIND | 15.41 (5.1) | 11.82 (4.2)* |
| QoL-AD | 35.65 (6.4) | 37.35 (5.3) |
| GDS-15 | 4.06 (3.6) | 3.47 (2.2) |
| Proxy-reported (N=32) |  |  |
| QoL-AD (proxy) | 34.53 (4.9) | 36.29 (5.0) |
| QoL-AD (combined) | 35.42 (5.4) | 37 (4.2) |
| HCS (proxy) | 8.13 (4.7) | 9.88 (5.1) |

**Table A.2** Types of Dementia Diagnoses. Values are Numbers (Percentages) Unless Stated Otherwise

| **Type of dementia** | **Total**  **(N=34)** | **V-iCST**  **(n=17)** | **TAU**  **(n=17)** | |
| --- | --- | --- | --- | --- |
| Alzheimer's Disease | 20 (58.8) | 9 (26.5) | 11(32.4) | |
| Vascular Dementia | 7 (20.6) | 4 (11.8) | 3 (8.8) | |
| Mixed Dementia | 2 (5.9) | 1 (2.9) | 1 (2.9) | |
| Dementia with Lewy Bodies | 4 (2.9) | 0 (0.00) | 1 (2.9) | |
| Frontotemperal Dementia | 2 (5.9) | 1 (2.9) | 1 (2.9) | |
| Posterior Cortical Atrophy | 2 (5.9) | 2 (5.9) | | 0 (0.0) |

**Table A.3** Interview questions mapped onto the consolidated framework of implementation research (CFIR)

| CFIR domains | Questions used in interview guide |
| --- | --- |
| Intervention characteristics | - How did you find the sessions you had with…? - Were the sessions helpful for you? In what way? - How did you find having the sessions online via Zoom? - Is there anything you would change about V-iCST? |
| Outer setting | - How did you find supporting the PwD to access the sessions? (Carer question) - How did you find having the sessions online via Zoom? (prompts: ‘anything that helped’; ‘would you have preferred online or in person sessions’) |
| Inner setting | - How did you find having the sessions online via Zoom? (prompt: ‘anything good or bad about having sessions online’) - Did you notice any impact of the V-iCST on the PwD? Is so, what? (Carer question) |
| Characteristics of individuals | - How did you find having the sessions online via Zoom? - How did you find supporting the PwD to access the sessions? (Carer question) - What did you think of the different activities? (prompt: were you able to understand the activities) |
| Process of implementation | - Is there anything you would change about V-iCST? - How did you find supporting the PwD to access the sessions? (prompt: anything you think would be helpful to do differently?) (Carer question) |

### **Table A.4** Comparison of the V-iCST and TAU Group Changes from Baseline and at 9 weeks, Without Adjustments (Imputed Data)

|  | **V-iCST at 9 weeks (n=17)** | **TAU at 9 weeks (n=17)** | **V-iCST change from baseline (n=17)** | **TAU change from baseline (n=17)** | **Difference in scores (95% CI), p-value** | |
| --- | --- | --- | --- | --- | --- | --- |
| Self-reported (N=34) |  |  |  |  |  |  |
| ADAS-Cog | 12.03 (11.6) | 17.6 (11.0) | -3.76 (8.59) | -0.82 (7.81) | -2.94 (-8.75; 2.87) | 0.308 |
| MoCA-BLIND | 14.9 (4.52) | 12.0 (6.39) | -0.56 (3.32) | 0.10 (3.16) | -0.66 (-2.89; 1.56) | 0.545 |
| QoL-AD (self-reported) | 37.4 (6.01) | 39.3 (4.21) | 1.72 (4.92) | 1.90 (4.82) | -0.18 (-3.59; 3.24) | 0.916 |
| GDS-15 | 3.37 (4.03) | 3.11 (3.93) | -0.69 (2.67) | -0.36 (2.62) | 0.89 (-1.78; 3.56) | 0.499 |
| Proxy reported (N=32) |  |  |  |  |  |  |
| QoL-(combined) | 36.9 (4.17) | 38.5 (3.87) | 1.45 (3.91) | 1.52 (4.31) | -0.06 (-2.93; 2.81) | 0.964 |
| QoL-AD (proxy) | 34.9 (5.42) | 37.0 (5.29) | 0.35 (3.93) | 0.74 (4.23) | -0.38 (-3.27; 2.50) | 0.786 |
| HCS (proxy) | 9.38 (6.13) | 9.40 (5.75) | 1.25 (3.18) | -0.48 (3.56) | 1.73 (-0.75; 4.21) | 0.163 |

**Table A.5** Comparison of the V-iCST and TAU group changes from baseline and at 9 weeks, adjusting for baseline outcome measures (observed data)

|  | **9 weeks** | | | | **Change from baseline** | | | | **Difference in scores (95% CI), p-value** | |
| --- | --- | --- | --- | --- | --- | --- | --- | --- | --- | --- |
|  | **Missing** | **V-iCST at 9 weeks** | **Missing** | **TAU at 9 weeks** | **Missing** | **V-iCST change from baseline** | **Missing** | **TAU change from baseline** | |  |
| ADAS-Cog | 3 | 14.4 (3.07) | 1 | 15.5 (3.06) | 3 | -1.676 (3.05) | 1 | -0.499 (3.05) | -1.18 (-3.50; 1.14) | 0.308 |
| MoCA-BLIND | 3 | 13.2 (2.49) | 1 | 13.6 (2.47) | 3 | -0.415 (2.58) | 1 | -0.136 (2.56) | -0.279 (-2.30; 1.74) | 0.779 |
| QoL-AD (self-reported) | 3 | 37.6 (2.97) | 1 | 39.0 (2.97) | 3 | 0.295 (2.97) | 1 | 1.74 (2.97) | -1.45 (-3.69; 0.79) | 0.197 |
| GDS-15 | 3 | 3.52 (1.41) | 1 | 3.04 (1.41) | 3 | 0.253 (1.39) | 1 | -0.158 (1.39) | 0.411 (-0.63; 1.46) | 0.426 |
| QoL-AD (combined) | 5 | 37.2 (2.88) | 1 | 38.4 (2.88) | 5 | 0.226 (2.88) | 1 | 1.37 (2.88) | -1.15 (-3.42; 1.12) | 0.308 |
| QoL-AD (proxy) | 4 | 35.6 (3.56) | 1 | 36.6 (3.55) | 4 | -0.139 (3.56) | 1 | 0.864 (3.45) | -1.00 (-3.76; 1.75) | 0.460 |
| HCS (proxy) | 4 | 10.0 (3.10) | 1 | 8.15 (3.09) | 4 | 1.45 (3.10) | 1 | -0.431 (3.09) | 1.88 (-0.53; 4.29) | 0.120 |

**Table A.6** Summary of fidelity ratings per facilitator

| **Facilitators/ PIN** | **Total number of sessions** | **Missing** | **Not applicable** | **Mean** | **SD** | **Range** | | **Mean %** | **Fidelity rating** |
| --- | --- | --- | --- | --- | --- | --- | --- | --- | --- |
|  |  |  |  |  |  | **Max** | **Min** |  |  |
| **A** |  |  |  |  |  |  |  |  |  |
| 4 | 10 | 4 | 0 | 26.60 | 1.51 | 28.00 | 22.00 | 76% | Moderate |
| 7 | 7 | 7 | 1 | 25.86 | 1.86 | 28.00 | 24.00 | 74% | Moderate |
| 14 | 11 | 3 | 0 | 29.55 | 3.27 | 34.00 | 24.00 | 84% | High |
| 16 | 14 | 0 | 2 | 28.67 | 3.25 | 34.00 | 19.00 | 82% | High |
| 18 | 14 | 0 | 1 | 29.79 | 3.02 | 35.00 | 23.00 | 85% | High |
| 19 | 12 | 2 | 2 | 31.42 | 2.71 | 34.00 | 24.00 | 90% | High |
| 23 | 5 | 9 | 0 | 29.00 | 2.83 | 32.00 | 19.00 | 83% | High |
| 25 | 12 | 2 | 0 | 29.33 | 3.75 | 35.00 | 24.00 | 84% | High |
| **Subtotal** | 85 | 27 | 6 | 28.82 | 3.28 | 35.00 | 19.00 | 82% | Moderate |
| **B** |  |  |  |  |  |  |  |  |  |
| 2 | 6 | 8 | 2 | 28.00 | 1.41 | 30.00 | 26.00 | 80% | High |
| **Subtotal** | 6 | 8 | 2 |  |  |  |  |  |  |
| **C** |  |  |  |  |  |  |  |  |  |
| 39 | 13 | 1 | 4 | 30.15 | 2.70 | 34.00 | 26.00 | 86% | High |
| **Subtotal** | 13 | 1 | 4 |  |  |  |  |  |  |
| **D** |  |  |  |  |  |  |  |  |  |
| 9 | 14 | 0 | 22 | 25.43 | 3.79 | 31.00 | 20.00 | 73% | Moderate |
| 13 | 14 | 0 | 22 | 25.50 | 3.35 | 30.00 | 20.00 | 73% | Moderate |
| 38 | 14 | 0 | 20 | 24.07 | 3.45 | 31.00 | 18.00 | 69% | Moderate |
| **Subtotal** | 42 | 0 | 64 |  |  |  |  |  |  |
| **E** |  |  |  |  |  |  |  |  |  |
| 33 | 13 | 1 | 8 | 23.92 | 2.33 | 27.00 | 20.00 | 68% | Moderate |
| 36 | 14 | 0 | 7 | 23.71 | 3.93 | 30.00 | 16.00 | 68% | Moderate |
| **Subtotal** | 27 | 1 | 15 |  |  |  |  |  |  |
| **F** |  |  |  |  |  |  |  |  |  |
| 13 | 14 | 0 | 1 | 28.00 | 1.91 | 33.00 | 27.00 | 88% | High |
| 32 | 14 | 0 | 1 | 30.64 |  |  |  |  |  |
| **Subtotal** | 28 | 0 | 2 | 29.32 |  |  |  |  |  |
| **TOTAL** | 188.00 | 37 | 93 | 27.45 | 3.9 | 35 | 26 | 78% | Moderate |

**Figure. A.1. CONSORT 2010 checklist of information to include when reporting a randomised trial**

| Section/Topic | Item No | Checklist item | Reported on page No |
| --- | --- | --- | --- |
| Title and abstract | | | |
|  | 1a | Identification as a randomised trial in the title | 1 |
|  | 1b | Structured summary of trial design, methods, results, and conclusions (for specific guidance see CONSORT for abstracts) | 1 |
| Introduction | | | |
| Background and objectives | 2a | Scientific background and explanation of rationale |  |
|  | 2b | Specific objectives or hypotheses |  |
| Methods | | | |
| Trial design | 3a | Description of trial design (such as parallel, factorial) including allocation ratio | 2 |
|  | 3b | Important changes to methods after trial commencement (such as eligibility criteria), with reasons | 3 |
| Participants | 4a | Eligibility criteria for participants | 3 |
|  | 4b | Settings and locations where the data were collected | 3 |
| Interventions | 5 | The interventions for each group with sufficient details to allow replication, including how and when they were actually administered | 4 |
| Outcomes | 6a | Completely defined pre-specified primary and secondary outcome measures, including how and when they were assessed | 5 |
|  | 6b | Any changes to trial outcomes after the trial commenced, with reasons | 5 |
| Sample size | 7a | How sample size was determined | 4 |
|  | 7b | When applicable, explanation of any interim analyses and stopping guidelines | N/A |
| Randomisation: |  |  |  |
| Sequence generation | 8a | Method used to generate the random allocation sequence | 4 |
|  | 8b | Type of randomisation; details of any restriction (such as blocking and block size) | 4 |
| Allocation concealment mechanism | 9 | Mechanism used to implement the random allocation sequence (such as sequentially numbered containers), describing any steps taken to conceal the sequence until interventions were assigned | 4 |
| Implementation | 10 | Who generated the random allocation sequence, who enrolled participants, and who assigned participants to interventions | 4 |
| Blinding | 11a | If done, who was blinded after assignment to interventions (for example, participants, care providers, those assessing outcomes) and how | 4 |
|  | 11b | If relevant, description of the similarity of interventions | 4 |
| Statistical methods | 12a | Statistical methods used to compare groups for primary and secondary outcomes | 6 |
|  | 12b | Methods for additional analyses, such as subgroup analyses and adjusted analyses | 6 |
| Results | | | |
| Participant flow (a diagram is strongly recommended) | 13a | For each group, the numbers of participants who were randomly assigned, received intended treatment, and were analysed for the primary outcome | 7, Figure 1 |
|  | 13b | For each group, losses and exclusions after randomisation, together with reasons | 7, Figure 1 |
| Recruitment | 14a | Dates defining the periods of recruitment and follow-up | 8 |
|  | 14b | Why the trial ended or was stopped | N/A |
| Baseline data | 15 | A table showing baseline demographic and clinical characteristics for each group | 8 |
| Numbers analysed | 16 | For each group, number of participants (denominator) included in each analysis and whether the analysis was by original assigned groups | 7, 11-13 |
| Outcomes and estimation | 17a | For each primary and secondary outcome, results for each group, and the estimated effect size and its precision (such as 95% confidence interval) | 7, 11-13 |
|  | 17b | For binary outcomes, presentation of both absolute and relative effect sizes is recommended | 11-13 |
| Ancillary analyses | 18 | Results of any other analyses performed, including subgroup analyses and adjusted analyses, distinguishing pre-specified from exploratory | 12-13 |
| Harms | 19 | All important harms or unintended effects in each group (for specific guidance see CONSORT for harms) | 12 |
| Discussion | | | |
| Limitations | 20 | Trial limitations, addressing sources of potential bias, imprecision, and, if relevant, multiplicity of analyses | 14 |
| Generalisability | 21 | Generalisability (external validity, applicability) of the trial findings | 15-16 |
| Interpretation | 22 | Interpretation consistent with results, balancing benefits and harms, and considering other relevant evidence | 15-16 |
| Other information | | |  |
| Registration | 23 | Registration number and name of trial registry | 3 |
| Protocol | 24 | Where the full trial protocol can be accessed, if available | N/A |
| Funding | 25 | Sources of funding and other support (such as supply of drugs), role of funders | 17 |
